# Laparoscopic transabdominal preperitoneal (TAPP) groin hernia repair using n-butyl-2-cyanoacrylate (Liquiband®Fix8™) for mesh fixation and peritoneal closure: learning experience during introduction into clinical practice

**DOI:** 10.1007/s10029-018-1861-6

**Published:** 2018-11-30

**Authors:** P. Wilson, L. Hickey

**Affiliations:** 1 University Hospitals of Morecambe Bay NHS Foundation Trust, Lancaster, UK; 2 Department of General Surgery, Royal Lancaster Infirmary, Lancaster, LA1 4RP UK

**Keywords:** TAPP repair, Cyanoacrylate glue, Mesh fixation, Peritoneal closure

## Abstract

**Introduction:**

In a single centre evaluation of a novel hernia repair device, 200 consecutive patients underwent 247 laparoscopic (TAPP) groin hernia repairs (47 bilateral) using *n*-butyl-2-cyanoacrylate (Liquiband**®**Fix8™) for mesh fixation and peritoneal closure over a 2-year period by a single experienced laparoscopic surgeon.

**Patients and methods:**

All groin hernia patients requiring TAPP repair were included in the study: Inguinal 142, Femoral 14, Spigelian 4, and Inguinal disruption 40. A retrospective review of the data was performed. There were 161 males and 39 females, mean age 55 years (range 20–89 years). Mesh fixation was successful in all 247 TAPP repairs, and 90% of patients had a successful peritoneal closure using the device (20 patients required the use of conventional tacks to complete closure).

**Follow-up:**

Patients were followed up with an out-patient visit at 6 weeks post-op, followed by a Patient Initiated Follow Up programme, and a final Telephone follow-up. To date all patients have completed 1 year of follow-up, and 70% of patients 2 years of follow-up (median 29 months, range 14 to 40 months).

**Results:**

There were very few procedure-related adverse events: groin seromas 6 (2.4%), port site bleeding 2 (0.3%), port site hernia 2 (0.3%), and only 1 groin hernia recurrence (0.4%). Prospective surgeon scoring of satisfaction for mesh fixation, peritoneal closure, and device clogging was favourable and increased following the initial learning phase. Changes in the device design during the study period improved the efficacy of the device significantly.

**Conclusion:**

This retrospective study shows that mesh fixation and peritoneal closure using the Liquiband®Fix8™ device is feasible, safe, practical, and is easy to learn.

## Introduction

Inguinal hernia repair is the most frequently performed operation in the United States [1] and in Europe [2]. Repair methods include open eg Lichtenstein or laparoscopic using a mesh fixated with tissue-penetrating methods (sutures, staples, tacks) or non-penetrating methods such as fibrin sealant, or no fixation [1, 3–7]. With unfixated mesh, higher mesh mobility, lower tensile strength, and increased risk of recurrence have been reported [8, 9].

Mesh fixation and peritoneal closure during laparoscopic groin hernia repair using tissue penetrating methods such as tacks can be associated with not insignificant morbidity including: vascular, nerve, muscle, and visceral injury, and may result in an increase in acute and chronic pain issues [10–13]. Complications associated with tacking techniques have been experienced by the author (Table 1).

**Table 1:** Complications associated with the use of tacks [ProTack™ (Covidien), AbsorbaTack™ (Covidien), and Securestrap™ (Ethicon)] for mesh fixation and peritoneal closure [Author’s experience in 3000 TAPP repairs (unpublished observations)]

Complication	Number of patients
Inferior epigastric vessel injury	32
Post-op haematoma	11
Surgical reintervention	4
Femoral nerve injury	1
Small bowel obstruction (prolapse into peritoneal gap due to failure of peritoneal closure)	2
Small bowel fistula (adherence to tacks)	1
Colonic fistula (adherence to tacks)	2
Chronic pain (muscle/nerve injury)	27
Injury to operating surgeon/assistant (tack penetration through to surface of abdominal wall)	3

Self-fixating mesh [ProGrip™ (Covidien)] can reduce these risks [14, 15], as can a sutured closure of the peritoneum, but is associated with a significant increase in operative time. Non-penetrative fixation of mesh with glue such as Fibrin has been shown to be safe and is associated with reduced postoperative pain [16–19], but cannot be used for peritoneal closure [3]. *N*-2-butyl cyanoacrylate (nBCA) glue, which has an increased strength compared to fibrin, however, is capable of not only mesh fixation, but peritoneal closure [20–22].

Kukleta et al. published a series of more than 1300 TAPP procedures with mesh fixations using nBCA [23]. They established a precise fixation technique with a volume of up to 4 microlitres per square centimetre, showing excellent mesh integrity without a single mesh or wound infection. He also demonstrated that the excellent biocompatibility of nBCA was sufficiently tested and that the glue permits an excellent stability achievable after only a few seconds. Mittermair et al. were the first to use LiquiBand Fix8™ (*n*-butyl-2-cyanoacrylate) for peritoneal closure [21]. From their experience, TAPP surgery using nBCA is suitable for mesh fixation and closure of the peritoneal defect. Dauser et al. performed a prospective study and have shown that non-penetrating fixation of mesh during TAPP repair using LiquiBand Fix8™ laparoscopic fixation device containing nBCA is highly effective [22]. They found closure of the peritoneum using exclusively nBCA to be safe and feasible.

The author who had been using tack fixation exclusively for both mesh fixation and peritoneal closure during TAPP repairs over a 20-year period, with not insignificant morbidity (Table 1), had explored alternative techniques to replace tacking to reduce morbidity. Self-fixating mesh [ProGrip™ (Covidien)] and suturing techniques were employed for a period of time, but were found to be associated with a significant increase in operative time compared to tacking. The large TAPP workload of the surgical unit required a simple, practical, safe, and less time-consuming technique for mesh fixation and peritoneal closure, and which was required to be non penetrative. In 2015 the Liquiband Fix8™—nBCA device was trialed and introduced into clinical practice in the unit.

## Study aims

The primary aim of this study was to ensure that this novel device (Liquiband**®**Fix8™) was safe and practical to use in clinical practice in routine TAPP repair for mesh fixation and peritoneal closure.

This would involve patient follow-up to monitor for adverse events including hernia recurrence, chronic pain, and episodes of small bowel obstruction (which might indicate failure of peritoneal closure, or adhesion formation related to the peritoneal closure with glue).

Secondary aims included its efficacy, ease of use, and learning curve, addressed by prospectively scoring the device for both mesh fixation and peritoneal closure.

## Patients and methods

The device was introduced into clinical practice in the author’s institution in April 2015, and replaced the previous tacking device [AbsorbaTack™(Covidien)] for mesh fixation and peritoneal closure during TAPP repairs.

All patients requiring TAPP repair, for a variety of hernias, were included in this study (inguinal hernia, femoral hernia, Spigelian hernia, Inguinal disruption). There were no exclusions. Inguinal hernias included direct (M1–3) and indirect (L1–3) defects (European Hernia Society Classification) [24]. Procedures were carried out by a single experienced laparoscopic surgeon—with an interest in abdominal wall hernia surgery, in a district general hospital.

### Device description

**Fig. 1:**
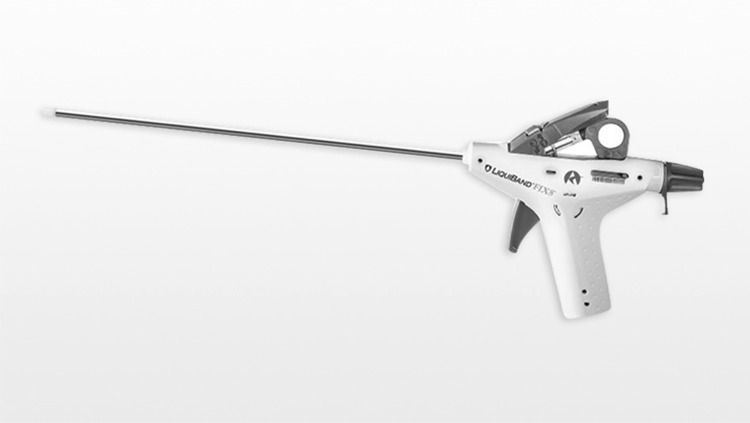
Liquiband Fix8™ Device

The Liquiband**®**Fix8™ device (Advanced Medical Solutions Ltd) is a non-penetrating laparoscopic fixation device. It has a 5 mm-diameter rigid delivery system with a trigger action (Fig. 1). The trigger fires a 12.5- mg (0.01 ml) aliquot of anchor solution (100% *n*-butyl-2-cyanoacrylate), and has 33 shots. There is a simple 6-stage preparation and priming process. The anchor solution setting time, once deployed from the device, is 5–10 s. The device has been available since May 2014. Developments in the device design have been facilitated to improve the tip—to reduce clogging of the device (April 2016) and the addition of D&C No. 2 Violet dye (August 2016) have improved visibility of the anchor solution. In 2018 the device was modified further to increase the number of shots of anchor solution from 33 to 44.

### Surgical technique

All patients received antibiotic prophylaxis with IV Gentamicin 160 mg and Metronidazole 500 mg. All TAPP repairs were carried out under general anaesthesia, using a standard 3-port technique (10 mm sub-umbilical and 2 lateral 5 mm ports) with CO_2_ pneumoperitoneum at 15 mmHg. The patient was tilted into the head down position. Following peritoneal incision, preperitoneal dissection was carried out using a standard technique. The medial extent of the dissection was 1 cm medial to the symphysis pubis, to allow sufficient overlap for direct (medial) defects. The lateral extent of the dissection was sufficient to ensure that the internal (deep) ring lay at the centre of the long axis of the 15 × 10 cm mesh used for the repair. This allowed sufficient lateral overlap for indirect (lateral) defects. A standard 15 × 10 cm polyester mesh [Parietex™ 3D Hydrophilic (Covidien)] was placed into the preperitoneal space and fixed with Liquiband Fix8™ superiorly with 5 anchors, medially over the pubis and pectineal ligament with 3 anchors, over the inferior epigastric vessels with 2 anchors, and over the floor (triangles of doom and pain) with 4 anchors. The defect was also encircled with anchors. In the Spigelian hernia repairs a similar size mesh was used with dissection sufficient to allow the 15 × 10 cm mesh to be centred on the Spigelian defect. The mesh was the secured with Liquiband Fix8™ along its superior border with 5 anchors, inferiorly with 5 anchors, and the defect then encircled with anchors. Peritoneal closure was carried out using Liquiband Fix8™ facilitated by dropping the intraabdominal pressure to 8 mmHg, and leveling the patient, to relieve tension on the closure. If the peritoneal closure was deemed ‘difficult’ due to a ‘wet field’ or ‘heavy peritoneum’ (sliding hernia), conventional tacks (AbsorbaTack™) were used to supplement and complete closure of the peritoneum. Bupivacaine 0.25% 20 ml was injected laparoscopically into the preperitoneal space via a laparoscopic gallbladder aspiration needle. Pneumoperitoneum was evacuated and then reinflated to check continuity of the peritoneal closure prior to port removal. Wounds were infiltrated with a further 20 ml 0.25% bupivacaine. Umbilical fascia was closed with 0 Vicryl**®** (Ethicon), and wounds closed using 3/0 subcuticular Vicryl Rapide™ (Ethicon) and Liquiband Flex™ (Advanced Medical Solutions) for wound dressing.

All patient’s procedures were carried out as day-case procedures (discharged within 6 h of surgery) and were given standard post-operative analgesia—codeine, paracetamol, and ibuprofen—unless contraindicated.

### Intraoperative device assessment

All patient’s procedures were scored prospectively for quality of mesh fixation and peritoneal closure using the Liquiband Fix8™ device using a scoring system devised by the author. A Surgeon Satisfaction Score (SSS, graded 1–4) for mesh fixation and for peritoneal closure was recorded by the senior surgeon (SSS 1 = poor, 2 = satisfactory, 3 = good, 4 = excellent). This Surgeon Satisfaction Score is defined in Table 2. Device clogging was also recorded (0 = none, 1 = minor ≤ 4 events, 2 = major ≥ 4 events). The number of Liquiband Fix8™ devices used was recorded. Any dropped anchor solution within the peritoneal cavity and onto small intestine or colon was also recorded.

**Table 2:** Surgeon Satisfaction Score (SSS) for mesh fixation and peritoneal closure—definitions

Grade	Quality	Definition
Surgeon satisfaction score (SSS)—Mesh fixation		
1	Poor	Additional tacks required for mesh fixation
2	Satisfactory	Glue mesh fixation at standard points (superior border)
3	Good	Glue mesh fixation including pubis and pectineal ligament
4	Excellent	Full extended glue mesh fixation including floor
Surgeon satisfaction score (SSS)—peritoneal closure		
1	Poor	Full tack closure/several tacks required for closure
2	Satisfactory	Glue peritoneal closure with a few additional tacks required for closure
3	Good	No tacks required, glue peritoneal closure with minor gaps
4	Excellent	Full glue peritoneal closure including holes with no gaps

The use of conventional tacks (AbsorbaTack™) to supplement or complete peritoneal closure (in the event of a ‘difficult’ glued peritoneal closure) was also recorded.

### Follow-up protocol

All patients received a follow-up appointment to be seen in the outpatient clinic by the senior surgeon at 6–8 weeks post operatively following discharge.

At that visit the patient was assessed with regard to postoperative recovery and adverse events, including wound healing, wound issues, port site swellings/lumps, groin lumps—seroma/haematoma/recurrence, post-operative pain, and return to normal activities.

After their review appointment all patients were offered a Patient Initiated Follow Up review for 12 months (and up to 24 months) following surgery. This involved written information as to the nature of Patient Initiated Follow Up, symptoms to report, and contact details to initiate a further follow-up appointment. The symptoms to report included the following:

- Persistent groin lump.
- Development of a new groin lump.
- Lumps developing at the sites of your incisions.
- Persistent groin pain.
- Development of new groin pain.
- Abdominal bloating and distension associated with vomiting.
- Change in bowel habit.

If the patient made contact to report a postoperative symptom a follow-up appointment was sent to the patient and they were reviewed in the outpatient clinic by the senior surgeon.

This follow-up protocol was a standard for all patients in the surgical unit undergoing TAPP repairs.

Patient electronic records were assessed retrospectively for patient and operative criteria, and follow-up data. Records were also assessed for patient readmission due to adverse postoperative events and reinterventions.

A telephone follow-up of all patients was carried out in August 2018. Patients were interrogated with regard to the PIFU symptoms (see above) and also with regard to consultations with their General Practitioner, and readmission to hospital.

## Results

200 consecutive patients underwent 247 TAPP repairs (47 bilateral) for groin hernias over a 25-month period (May 2015–June 2017). Patient characteristics and operative findings are shown in Table 3. Hernia type is also documented, but the hernia size was not recorded.

**Table 3:** Patient characteristics and operative findings of the 200 patients undergoing TAPP repairs

Characteristic/operative findings	
Sex	Male: 161, Female: 39
Age	Mean 55 years, Median 58 years, Range 20–89 years
Hernia side	Bilateral 47, Left unilateral 65, Right unilateral 88
Hernia type	Inguinal 142 (39 bilateral), Femoral 14 (4 bilateral), Spigelian 4, Inguinal disruption 40 (4 bilateral)
Recurrent hernias	24 (inguinal 21, femoral 2, Spigelian 1)
Elective/emergency	198 elective, 2 emergency
Comorbidities	46 patients: Hypertension 20, COPD 9, Anticoagulated 7, Cardiac disease 5, CVA/Parkinsons disease 3, Type II diabetes 3, Prostate cancer 3, Blood disorders 2
Simultaneous procedures	16 patients: Umbilical hernia repair 14, Cholecystectomy 1, Varicose vein stripping 1
Intraoperative findings	Inguinal Hernia patients–direct (M1–3) 108, indirect (L1–3) 84, incidental femoral hernias 17 Femoral Hernia patients–incidental inguinal hernia 5, incidental obturator hernia 3
Mesh used	Parietex™ 3D Hydrophilic mesh (Covidien) 199 patients (246 TAPP repairs) Surgimend™ (Integra) 1 patient (simultaneous cholecystectomy)
Length of stay	Day case procedures: 192 patients (96%)

### Mesh fixation

**Fig. 2:**
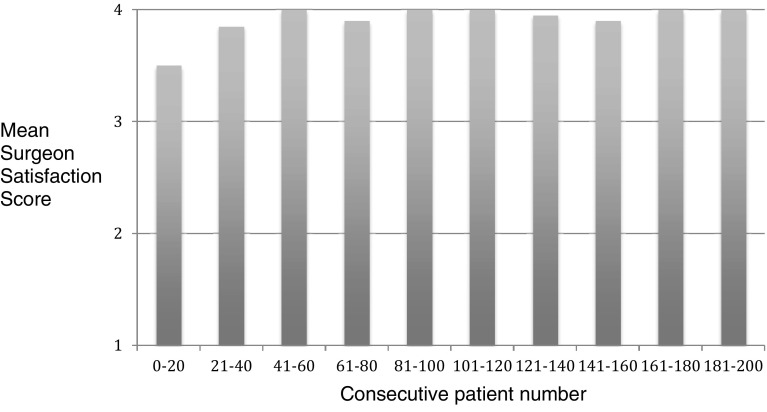
Mean Surgeon Satisfaction Scores for Mesh Fixation (1poor, 2 = satisfactory, 3 = good, 4 = excellent. See Table 2 for definition)

Mesh fixation was carried out in all 200 patients (247 TAPP repairs) and Surgeon Satisfaction Scores were good (18/200) or excellent (182/200) in all patients. 13 of the 18 patients (72%) who had dropped a point (good as opposed to excellent) were in the first 23 consecutive cases carried out (Fig. 2).

### Peritoneal closure

Peritoneal closure was carried out with Liquiband Fix8™ alone in 222 TAPP repairs (90%).

25 TAPP repairs (20 patients, 10%) required the use of tacks (AbsorbaTack™) to facilitate peritoneal closureצ4 partial use of tacks, 21 full tack peritoneal closure).

**Fig. 3:**
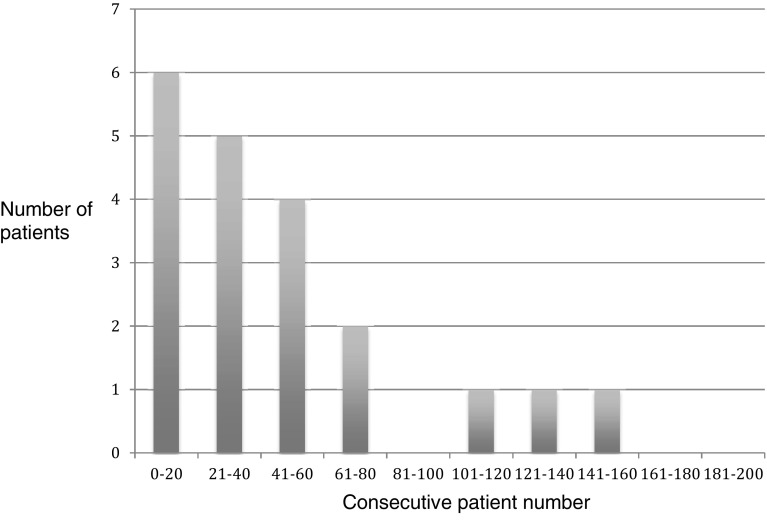
Patients requiring tacks to facilitate or supplement peritoneal closure

**Fig. 4:**
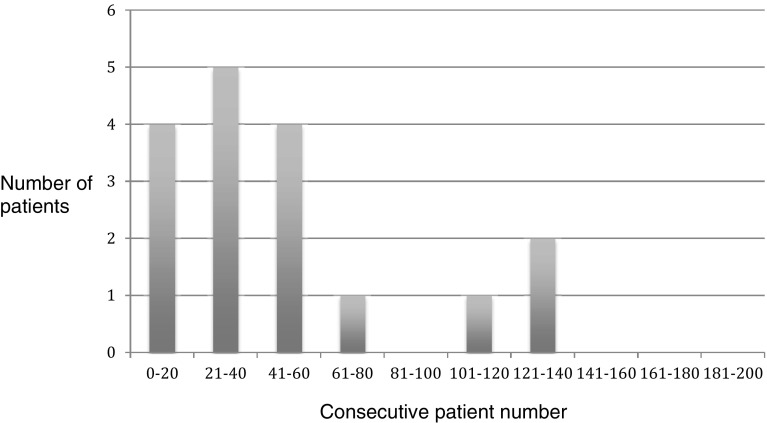
Patients with Surgeon Satisfaction Score for Peritoneal Closure—graded Poor (Full tack peritoneal closure/several tacks required for peritoneal closure)

15 out of these 20 patients (75%) requiring tack peritoneal closure/supplementation were in the first 60 consecutive cases performed (Fig. 3). Similarly, of the patients graded poor for peritoneal closure 13 out of 17 patients (76%) were in the first 60 consecutive cases performed (Fig. 4).

The reasons recorded for the use of tacks to complete the peritoneal closure was ‘wet field’ in 11 repairs, and ‘heavy peritoneum’ in 14 repairs.

**Fig. 5:**
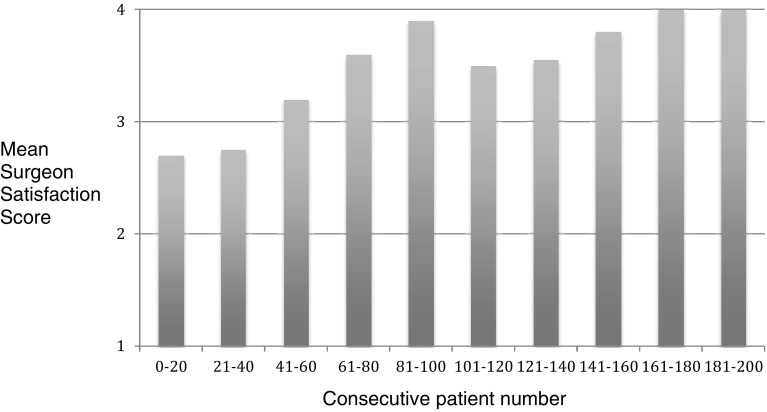
Mean Surgeon Satisfaction Scores for Peritoneal Closure (1 = poor, 2 = satisfactory, 3 = good, 4 = excellent. See Table 2 for definition)

Figure 5 shows the mean SSS for peritoneal closure in consecutive patients. There is a general trend of improvement in scores over the series.

### Device clogging

**Fig. 6:**
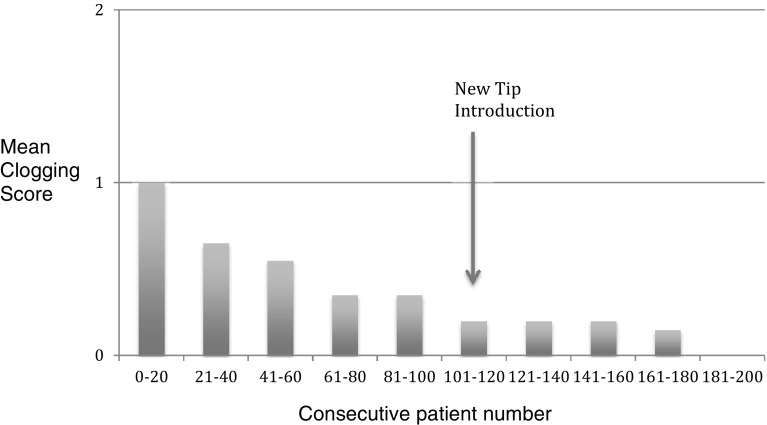
Mean Clogging Scores (0 = none, 1 = minor < 4 events, 2 = major > 4 events)

Figure 6 shows the mean clogging scores for the device over the course of time in the series of 200 patients. There was a steady trend of improvement. The modified device tip (designed to reduce clogging) was introduced at patient 111 in the series.

Major clogging of the device (> 4 clogging events) occurred in 11 patients (5.5%) using the early device (cases 1 to 110) only.

Minor clogging (< 4 clogging events) occurred in 48 patients (24%)—37/110 patients (34%) using the early device, and 11/90 (12%) using the modified tip device.

In 141 patients (71%) there was no device clogging.

**Fig. 7:**
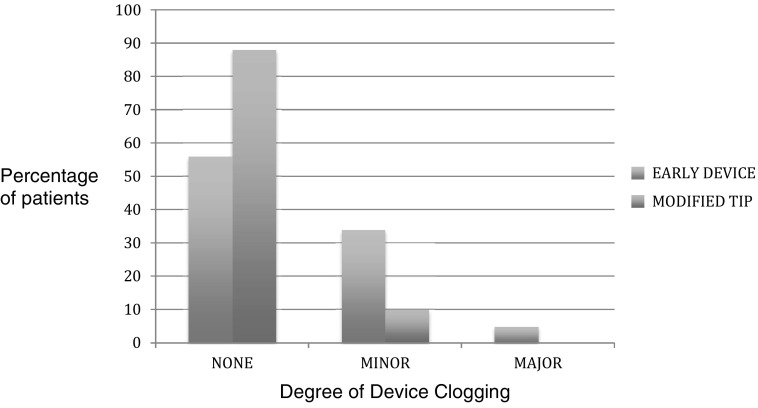
Clogging in the Early Device (Blue) and Modified Tip Device (Red) (none—no events, minor < 4 events, major > 4 events)

Figure 7 shows the clogging scores for the early device and the modified tip device.

### Number of liquiband Fix8™ devices used per repair

In 213 TAPP repairs (86%) a single device was used to complete mesh fixation and peritoneal closure.

In 31 repairs (13%) 2 devices were used.

In 1 repair 3 devices were used (patient receiving a Biological mesh – Surgimend™).

In 1 patient a single device was used for bilateral repairs for both mesh fixation and peritoneal closure.

### Dropped anchor solution

In 4 patients anchor solution from the device was inadvertently dropped onto the peritoneum, small intestine, or colon during peritoneal closure. In all of these patients the anchor solution, which had solidified, was partially retrieved (some minor fragments remained adherent to intestine).

In these patients no adverse events were observed intraoperatively, or in the postoperative follow-up period.

### Follow-Up

Ninety percent (179/200) of patients attended their initial 6- to 8-week outpatient follow-up visit and were then entered into the Patient Initiated Follow Up programme (PIFU).

Patient-initiated follow-up appointments were triggered in 10 patients (5.6%). These were related to: groin lump in 2 patients, port site lump in 1 patient, and chronic pain in 7 patients. Those patients requesting a follow-up for chronic pain were all following repair for inguinal disruption.

All patients with chronic groin pain following TAPP repair for inguinal disruption were reviewed at regular intervals in the outpatient clinic to monitor symptoms. In all patients postoperative pain resolved within 4 months.

There were no acute readmissions recorded following surgery, and no re-referrals from General Practitioners.

Telephone follow-up was performed in August 2018 in all patients and was successful in 197 of the 200 patients. Three patients were lost to follow-up due to inability to facilitate contact.

Follow-up:

- Range 14–40 months
- Median 29 months
- Mean 28 months
- Patients completing 1 year of follow-up: 197/197 (100%)
- Patients completing 2 years of follow-up: 137/197 (70%)

In the 197 patients completing the telephone follow-up there were no further groin hernia recurrences. All patients with groin seromas had complete resolution. All patients with chronic pain had full resolution of symptoms. No patients had developed symptoms of small bowel obstruction or change in bowel habit. There were no consultations with General Practitioners or hospital readmission related to small bowel obstruction. One patient reported an umbilical swelling related to a probable port site hernia which was asymptomatic. The patient was offered a review appointment in the outpatient clinic.

### Adverse events

Adverse events were seen in 13 patients (7%). These patients’ characteristics are shown in Table 4.

**Table 4:** Adverse events—patient characteristics

Adverse event	No. of patients	Age	Sex	Hernia type	Simultaneous procedures performed	Comorbidities/anticoagulants	Reintervention
Urinary retention	1	61	M	Inguinal-direct			
Surgical emphysema	1	70	F	Femoral-bilateral			
Groin seroma	6	71	M	Spigelian			
		72	M	Inguinoscrotal		AF/dabigatran	
		43	M	Inguinal-direct			
		59	M	Inguinal-direct			
		76	M	Inguinal-indirect	Cholecystectomy Biological mesh used		
		51	F	Femoral			
Groin hernia recurrence	1	63	M	Inguinal-indirect	Varicose vein stripping	Recurrence (indirect) at 8 months	Open Lichtenstein repair
Lateral port site haematoma	1	77	M	Inguinal-direct	Umbilical hernia repair	Prostate Ca	
Umbilical port site hernia	2	30	M	Inguinal-indirect			
		54	M	Inguinal-indirect			
Lateral port site haemorrhage	1	83	M	Inguinal-bilateral direct		Mechanical heart valve / warfarin	Laparoscopy-evacuation of haemoperitoneum

There was only one adverse event potentially attributable to the use Liquiband Fix8™ for mesh fixation—notably the one groin hernia recurrence.

There were no adverse events attributable to failure of peritoneal closure using Liquiband Fix8™ (no events related to small bowel obstruction, or intestinal fistulation).

### Discussion

The results show that mesh fixation and peritoneal closure with the device is safe, as shown by the lack of adverse events over the follow-up period (median 29 months), albeit, this is a relatively short follow-up. The single device-related adverse event, notably the patient with recurrence at 8 months, related to mesh migration is reassuring.

This adverse event may have had other contributory factors. The patient in question had a simultaneous procedure of varicose vein stripping following the TAPP repair which involved the same side and involved repositioning of the patient—Trendelenburg—with prolonged leg elevation. This may have influenced mesh slippage. The patient in question had recovered from the combined procedure very quickly and had required no postoperative analgesia, returning to a heavy physical workload at 4 days postop. The lack of postoperative pain certainly influenced this early return to work and did not impede the patient in carrying out heavy physical labour. This very early return to heavy physical duties may have influenced the prolapse of the mesh causing recurrence. The patient underwent a revisional procedure to repair the recurrence and at operation a significant indirect recurrence was apparent on open exploration of the inguinal canal. Recurrence had occurred due to the upper border of the mesh slipping downwards in a caudal direction. This is an unusual situation for recurrence following TAPP repair carried out using a penetrative tacking technique—which is most commonly related to the lower border of the mesh (which has not been fixed) lifting upwards allowing prolapse and recurrence underneath the lower border. The unusual form of recurrence in this patient may be related to the differences in technique of mesh fixation for a non-penetrative gluing technique compared to a penetrative tacking technique.

**Fig. 8:**
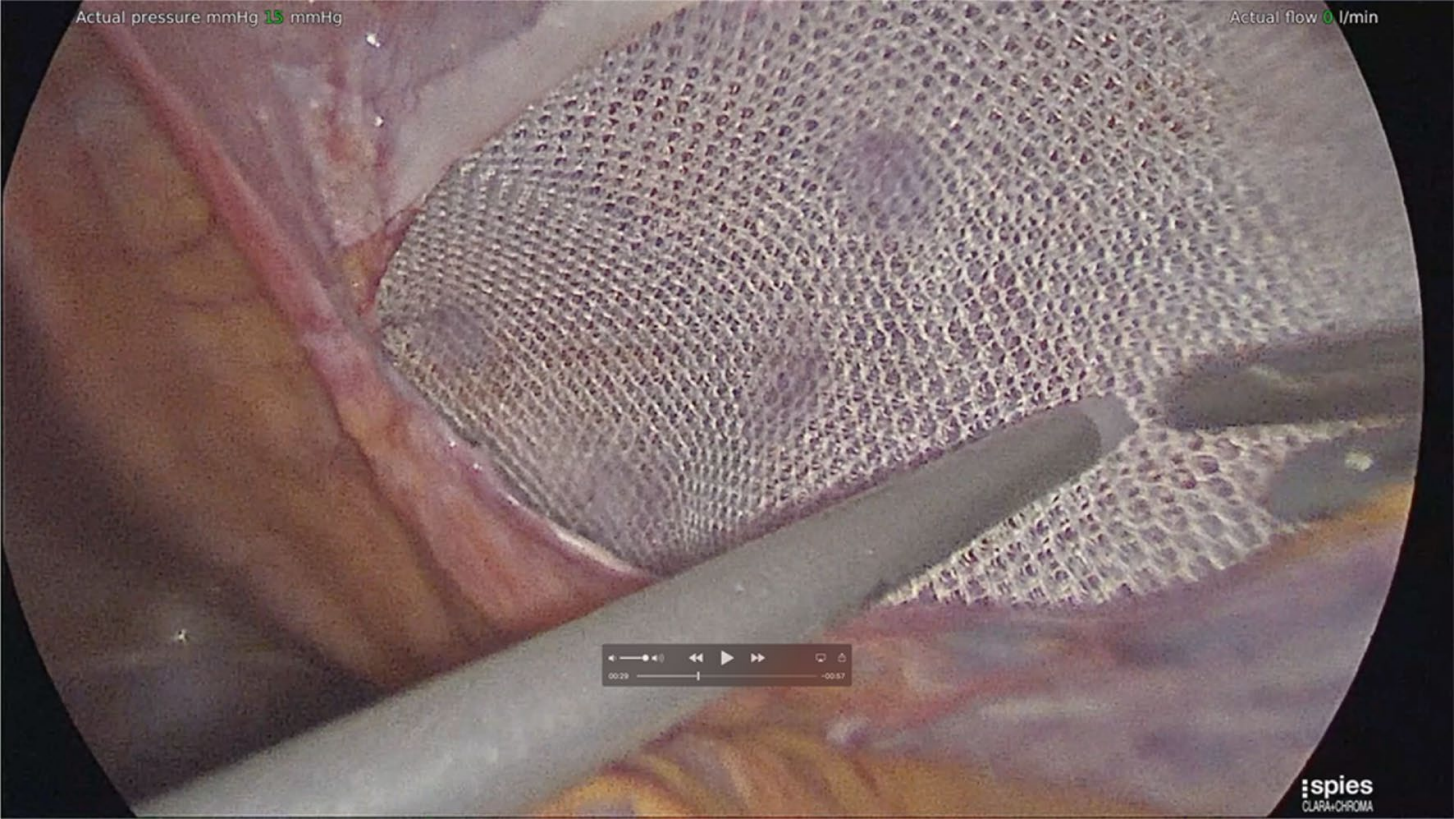
Mesh fixation using Liquiband Fix8™—Fixation over the ‘Triangle of Doom’ (External Iliac Vessels)

Mesh fixation using Liquiband Fix8™ (Fig. 8) not only involves anchoring the mesh on the traditional upper border, but also on the floor—over the external iliac vessels, and femoral nerve, over the inferior epigastric vessels, and over the pubis/pectineal ligament, and around the defect. This certainly gives a better fixation of areas which tacking techniques would not allow—due to injury to these structure and chronic pain issues. The degree of fixation over the pubis and pectineal ligament appears to be improved as more anchor points can be used in these areas. Potentially, this improved extended mesh fixation could translate into reduction in recurrence risk for TAPP repair. The non-penetrative gluing technique of mesh fixation would also have the potential to reduce inferior epigastric vessel injury and bleeding complications, chronic pain related to pubic stapalgia, nerve injuries, and muscle injury/bleeding which is the cause of much of the perioperative pain associated with penetrative tacking techniques. Similarly, during peritoneal closure using tacking techniques, one has to be aware (as in mesh fixation) of the position of the inferior epigastric vessels to avoid injury to these vessels with its associated bleeding risk and formation of haematomas. Tacking closure of the peritoneum during TAPP repair involves a further line of tacks (in close proximity to the line of tacks for upper border mesh fixation) with resultant muscle pain and potential for muscle bleeding and chronic pain. Potentially, mesh fixation and peritoneal closure with a non-penetrative gluing technique such as Liquiband Fix8™ should reduce the risk of adverse events and perioperative pain allowing earlier return to normal activities and reduced analgesic requirements. We have certainly seen a reduction in perioperative pain with the use of Liquiband Fix8™ compared to our previous tacking techniques. This was a very striking observation in our patients undergoing TAPP repair with Liquiband Fix8™. However, this was a subjective finding, and this study did not specifically look at peri-operative pain.

**Fig. 9:**
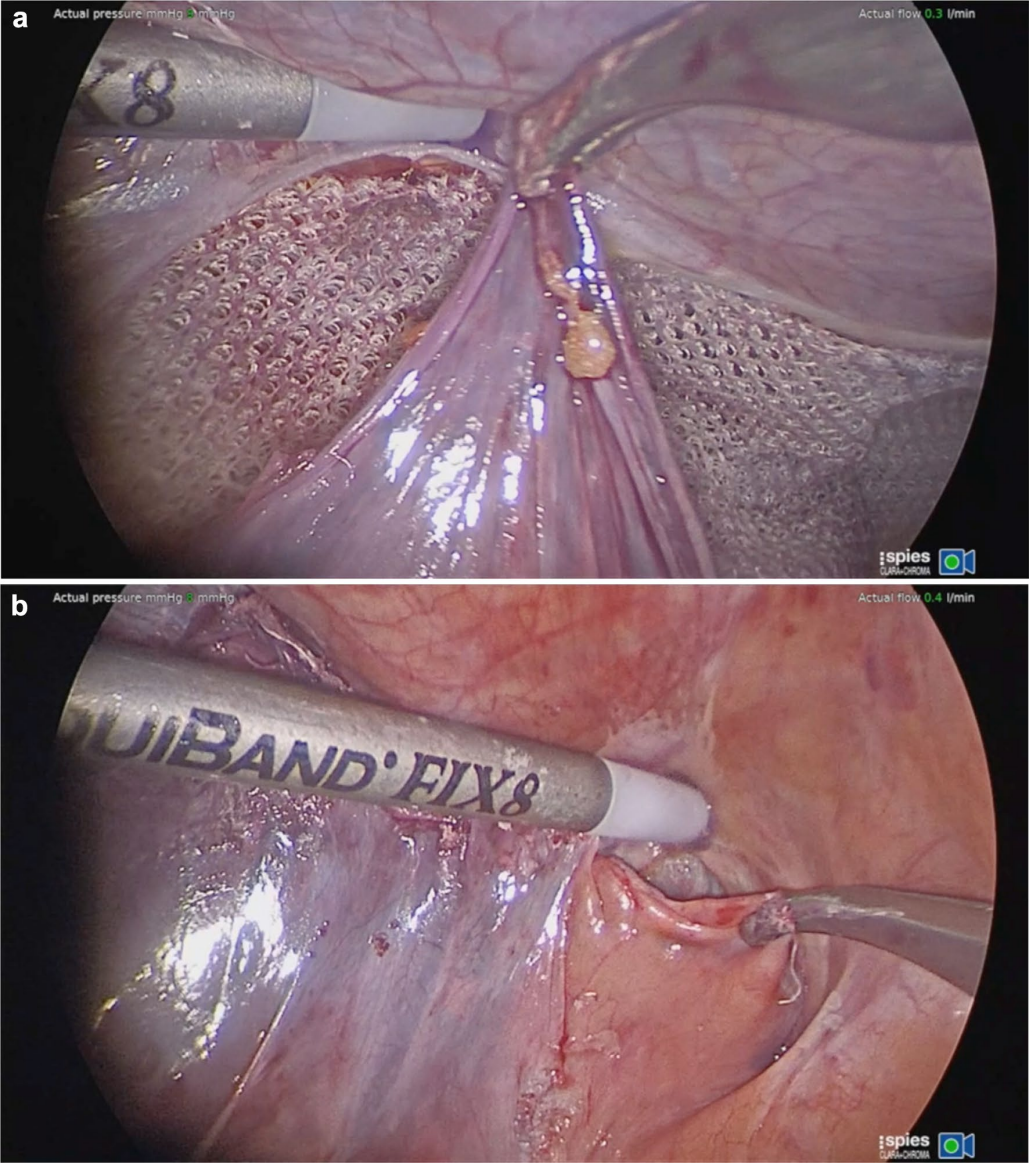
**a** Initial peritoneal closure using liquiband Fix8™—central fixation point. **b** peritoneal closure using liquiband Fix8™—Medial border

**Fig. 10:**
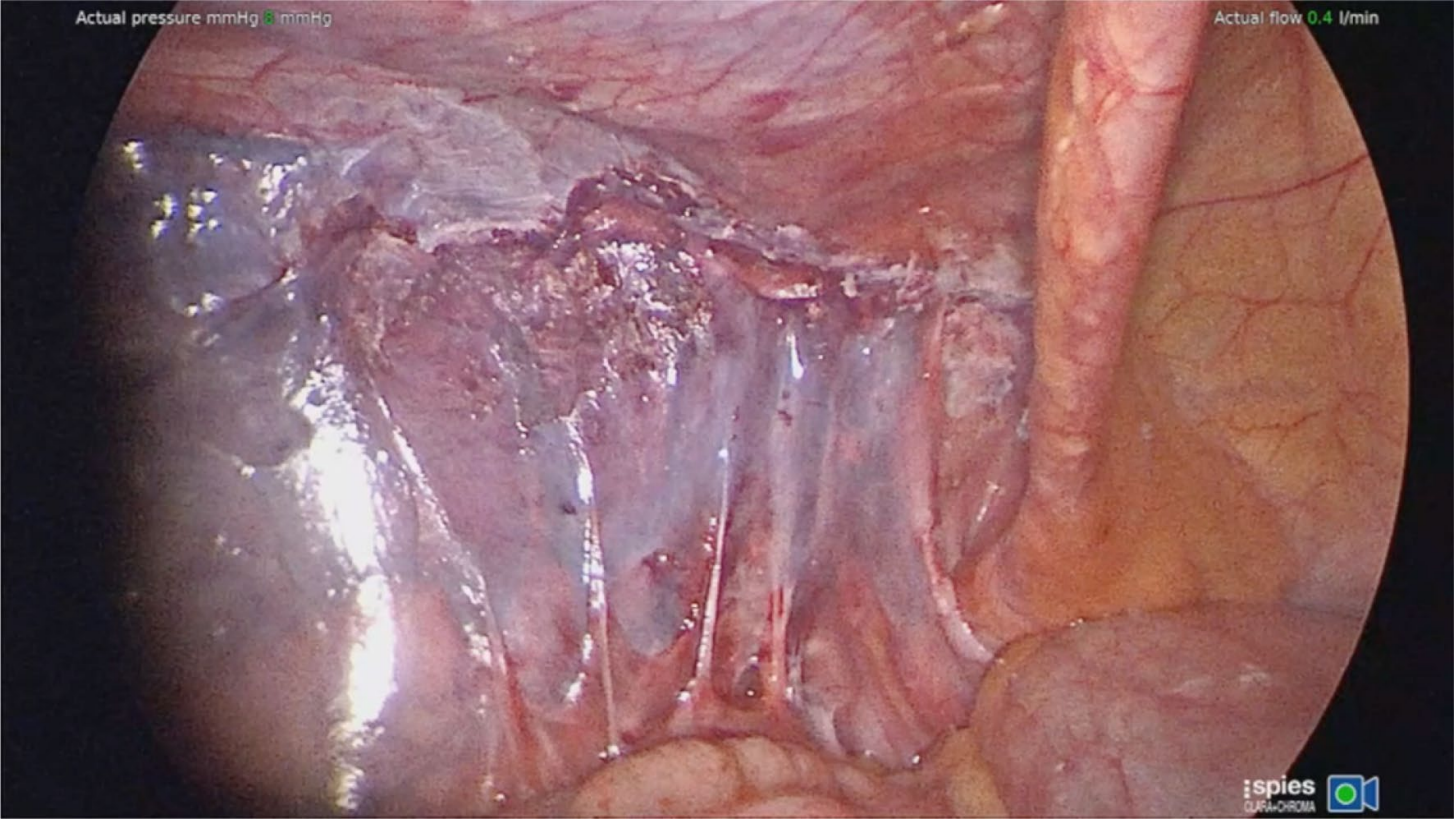
Completed peritoneal closure using liquiband Fix8™

There have been some concerns as to the safety of peritoneal closure using cyanoacrylates such as Liquiband Fix8™ as this is a relatively novel development. However, studies so far reported (albeit with relatively small numbers of patients) have shown evidence of its safety [21, 22]. In this study we have had no adverse events related to failure of peritoneal closure using the device. Failure of the peritoneal closure can be a serious adverse event associated with small bowel prolapsing into these gaps and becoming incarcerated and obstructed. It can also allow the mesh to become exposed to the small and large intestine which ultimately can produce fistulation. It has been our experience that the peritoneal closure using Liquiband Fix8™ (Fig. 9a, b) is far superior to that of the standard tacked peritoneal closure and is more equivalent to a sutured closure. The peritoneal closure using Liquiband Fix8™ appears more complete (Fig. 10), without gaps, and without exposed protruding fixation points. It would appear to be a more physiological closure, with the potential for reduced risk of small bowel, colonic, and omental adhesion. It is certainly very simple to close small and large holes in the peritoneum with Liquiband Fix8™ which have inadvertently been made during peritoneal dissection. These holes, if significant, would normally require closure using sutures due to their proximity to the floor of the dissection where tacking is risky. Sutured closure of these gaps and holes is time-consuming and laborious, and now can be simplified by using Liquiband Fix8™.

There are slight modifications to the operative technique for mesh fixation and peritoneal closure in using Liquiband Fix8™ compared to traditional tacks. We found these simple to learn and master, with a short learning curve. The simple scoring systems we used to assess our learning curve involved Surgeon Satisfaction Scores for mesh fixation and peritoneal closure. We observed that for mesh fixation using Liquiband Fix8™ the learning curve was very short (Fig. 2). Within the first 20 consecutive procedures carried out, our satisfaction scores, and confidence in using the device, reached a plateau. The learning curve for peritoneal closure was slightly longer, as this technique differs significantly compared to a tacking closure. This was assessed by Surgeon Satisfaction Scores (Figs. 4, 5). In Fig. 5 it can be observed that the plateau occurs after the first 80 cases. Those patients scored ‘poor’ for peritoneal closure (Fig. 4) can be seen to diminish to a plateau after 80 consecutive cases. The supplementary use of tacks to close the peritoneum in our study was not due to the failure of the device or gluing technique. This was related to a lack of confidence in using the device in challenging cases. The supplementary use of tacks diminished steadily over the course of the study and had reached a plateau after the first 80 consecutive cases as our confidence built in using the device. The use of supplementary tacks to complete peritoneal closure was predominantly in ‘difficult’ peritoneums—some of these were related to sliding hernias involving the sigmoid colon or caecum which created a weighty peritoneum with increased tension on the peritoneal closure. However, during our learning experience we found that minor modifications to technique allowed us to close the difficult peritoneums with ease. These modifications included: reduction in the CO_2_ pneumoperitoneal pressure from 15 mmHg down to 8 mmHg to reduce the tension on the peritoneal closure; leveling the patient position (from a head down position) and even putting the patient into a head up position which allowed gravity to assist in reducing the tension on the peritoneal closure. Another source of ‘difficult’ peritoneum to close and hence the use of supplementary tacks was the’wet’ peritoneum—that is minor blood staining of the peritoneal edge which occurred during the peritoneal dissection. This tended to cause the device tip to clog resulting in the necessity to remove the device to de-clog (using a fine needle). This was overcome during our learning phase by modifications made by Advanced Medical Solutions to the design of the Liquiband Fix8™ device tip (increasing the bore of the tip and its chamber size) following clinician feedback. We also learnt to reduce the ‘wetness’ of the field by placing small mastoid swabs into the peritoneal cavity and to mop the field dry. This also made a significant difference to the ease of peritoneal closure and the reduction of supplementary tacks. A further area of ‘difficult’ peritoneum to close was the ‘fatty’ peritoneum. The anchor solution of the Liquiband Fix8™ device performed less satisfactorily when trying to glue the fatty internal side of the lower peritoneal flap to the peritoneal surface of the upper peritoneal flap. We have learnt to invert the lower peritoneal flap in these cases which allows us to glue the peritoneal surface of both lower and upper peritoneal flaps together, avoiding glue application to ‘fatty’ areas. Many of these techniques were learnt in the course of our experience in the first 100 cases. We now feel confident with the device and use its strengths to our (and the patient’s) advantage. We no longer have difficulty closing the peritoneum with Liquiband Fix8™, even under some very challenging conditions, and feel confident with the device and its ability to effectively close the peritoneum.

Device clogging—that is clogging of the device tip stopping the issue of anchor solution was a feature of the device (albeit not a major problem) which was monitored during the study period. Clogging was related not only to device tip design but also related to use of the device itself. The company (Advanced Medical Solutions) did make changes to the device tip design (increased bore and chamber of the tip) during the study period which significantly reduced the clogging events (Figs. 6, 7). A further development in the design involved improving the visibility of the anchor solution. In the early devices the anchor solution was a clear solution which at times was difficult to see intraoperatively. The addition of a violet dye (D&C number 2 violet) greatly improved visibility of the anchor solution expressed from the device tip. This improved the ease of use of the device.

Further developments have recently been made in the device which have increased the number of ‘shots’ of anchor solution (to 44) which have improved the cost effectiveness of the device.

Whilst cost effectiveness was not studied in our series, the mean number of devices used per TAPP repair (one device) equates in cost to the use of tacking devices (The cost of a Liquiband Fix8™ device is similar to that of an AbsorbaTack™ device).

Our confidence in using the Liquiband Fix8™ device for mesh fixation and peritoneal closure in TAPP repair has allowed us to incorporate this device and the technique into our standard clinical practice in our unit. We no longer require the use of tacks for mesh fixation or peritoneal closure for TAPP repair.

It has also allowed us to extend its role for mesh fixation in laparoscopic intra-peritoneal onlay mesh (IPOM) repairs for incisional hernias.

## Conclusion

The use of *n*-butyl-2-cyanoacrylate (Liquiband**®**Fix8™) for mesh fixation and peritoneal closure in TAPP repair is safe, practical, and an effective alternative to tacking techniques.

The modified technique to use this device is simple to master, with a short learning curve.

The device is capable of an extended mesh fixation, in areas that are not appropriate with tacking or suturing techniques which may reduce the risk of hernia recurrence.

It also offers advantages in potential reduction in adverse events such as bleeding complications, nerve injury, and chronic pain associated with conventional tacking techniques.
